# Post-epizootic salamander persistence in a disease-free refugium suggests poor dispersal ability of *Batrachochytrium salamandrivorans*

**DOI:** 10.1038/s41598-018-22225-9

**Published:** 2018-02-28

**Authors:** Annemarieke Spitzen - van der Sluijs, Gwij Stegen, Sergé Bogaerts, Stefano Canessa, Sebastian Steinfartz, Nico Janssen, Wilbert Bosman, Frank Pasmans, An Martel

**Affiliations:** 1 Reptile, Amphibian & Fish Conservation Netherlands (RAVON), PO Box 1413, 6501 BK Nijmegen, The Netherlands; 2 Wildlife Health Ghent, Department of Pathology, Bacteriology and Avian Diseases, Faculty of Veterinary Medicine, Ghent University, Salisburylaan 133, B9820 Merelbeke, Belgium; 3 Lupinelaan 25, 5582 CG Aalst-Waalre, The Netherlands; 4 Technische Universität Braunschweig, Zoological Institute, Evolutionary Biology and Molecular Ecology, Mendelsohnstraße 4, 38106 Braunschweig, Germany; 5 Kaarskoopruwe 2, 6218 XA Maastricht, The Netherlands

## Abstract

Lack of disease spill-over between adjacent populations has been associated with habitat fragmentation and the absence of population connectivity. We here present a case which describes the absence of the spill-over of the chytrid fungus *Batrachochytrium salamandrivorans* (Bsal) between two connected subpopulations of fire salamanders (*Salamandra salamandra*). Based on neutrally evolving microsatellite loci, both subpopulations were shown to form a single genetic cluster, suggesting a shared origin and/or recent gene flow. Alpine newts (*Ichthyosaura alpestris*) and fire salamanders were found in the landscape matrix between the two sites, which are also connected by a stream and separated by no obvious physical barriers. Performing a laboratory trial using alpine newts, we confirmed that Bsal is unable to disperse autonomously. Vector-mediated dispersal may have been impeded by a combination of sub-optimal connectivity, limited dispersal ability of infected hosts and a lack of suitable dispersers following the rapid, Bsal-driven collapse of susceptible hosts at the source site. Although the exact cause remains unclear, the aggregate evidence suggests that Bsal may be a poorer disperser than previously hypothesized. The lack of Bsal dispersal between neighbouring salamander populations opens perspectives for disease management and stresses the necessity of implementing biosecurity measures preventing human-mediated spread.

## Introduction

Emerging infectious disease of wildlife are a leading cause of biodiversity loss worldwide^1^. Because successful mitigation of epizootics remains extremely challenging^2^, most recommended strategies for controlling disease impacts focus on creative local, context-specific solutions that minimize the spatial diffusion of pathogens, mostly through generally applicable biosafety measures and restrictions to trade and other human-mediated movements of wildlife^3,4^. Devising effective actions aimed at minimizing disease spread, that go beyond those general biosafety precautions, would require a better understanding of the dynamics of such spread and its preferential pathways. This type of information is especially vital at the early stages of an emerging disease invasion^5^. Dispersal abilities of pathogens, hosts and vectors (biotic and abiotic), the presence and role of barriers to dispersal, as well as stochastic processes that determine whether spread occurs or not, all need to be investigated^3,4^.

In northwestern Europe, the recently detected chytrid fungus *Batrachochytrium salamandrivorans* (hereafter: Bsal)^6^ has brought several populations of fire salamanders (*Salamandra salamandra*) to the brink of extinction within a short time frame of five years or less^6–10^. The fungus persists in natural systems and currently no viable solution is at hand to eliminate Bsal from infected wild populations or to reduce its impact^11^. Previous studies have hypothesized that Bsal should be able to spread rapidly, similarly to *B*. *dendrobatidis* (hereafter: Bd)^12^, thus posing a concrete risk of a novel amphibian pandemic^13,14^. On the other hand, Canessa *et al*.^11^ suggested that high mortality rates mean that Bsal-infected fire salamanders are generally unlikely to move long distances, although resistant spores and other hosts and vectors may still facilitate dispersion^10^.

In October 2013, a population of fire salamanders was discovered in the Netherlands in a marginal habitat site (hereafter referred to as Broek) located 800 m from the index-case of Bsal in Europe (Bunderbos)^6,7^. The landscape between the two sites consists of built-up areas alternating with agricultural land, lined with hedgerows and small forest patches and a partially underground stream that connects the two subpopulations (Broek and Bunderbos). In the absence of obvious physical barriers such as highways, invasion by Bsal in the newly discovered subpopulation and its ensuing total collapse were considered imminent. However, to date these events have not occurred and the Broek subpopulation remains apparently free from Bsal.

To clarify the potential factors determining this failure of Bsal spread between two neighbouring sites, we carried out field surveys at both the Broek and Bunderbos subpopulations to estimate fire salamander abundance, to quantify Bsal prevalence and infection loads, and to estimate the genetic differentiation and gene flow between the subpopulations. We also conducted a laboratory experiment with alpine newts (*Ichthyosaura alpestris*, a demonstrated Bsal vector sympatric with fire salamanders^10^), in which we determined the ability of Bsal to spread autonomously when host contact is physically impeded. We present the results of these analyses and discuss their implications for Bsal dispersal and potential mitigation strategies.

## Results

### Persistence of a stable Bsal-free salamander subpopulation in the vicinity of a Bsal outbreak site

**Figure 1:**
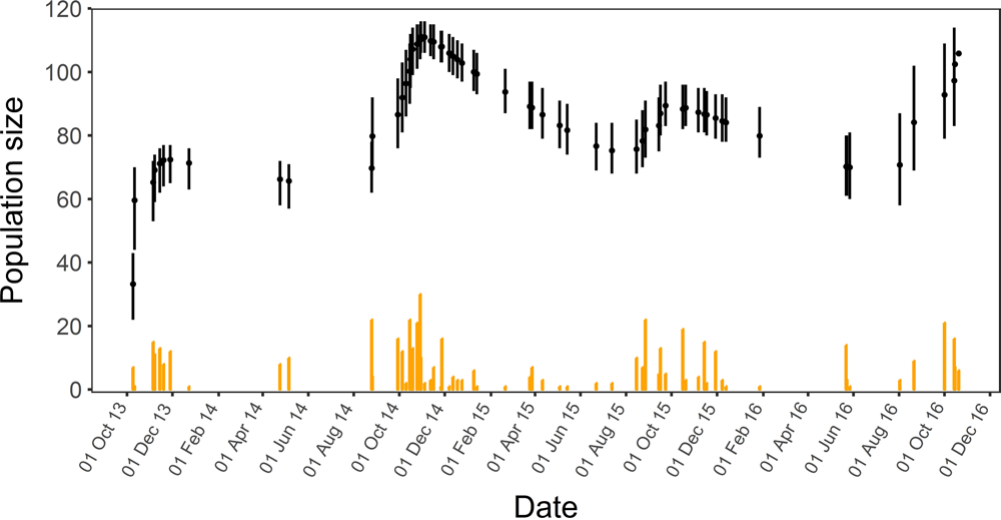
Population size of fire salamanders at the new site, estimated from open-population Jolly-Seber model. Bars indicate 95% credible intervals. Orange bars indicate the total count of captures on a given survey.

To quantify the Bsal prevalence and infection load we collected skin swabs from the ventral side of salamanders. In the Broek subpopulation, 176 unique fire salamanders were caught over 64 site visits for a total of 510 sightings. This included 139 adults, 33 sub-adults and 4 juveniles (considering the oldest age class of capture for each individual). Sex ratio was slightly biased towards males (78 M:69 F) although 29 animals could not be sexed with certainty. Individual fire salamanders were recaptured between 1 and 16 times (mean 2.9 times; median = 2 times); the majority of individuals (n = 71) were sighted once, and 35 animals were sighted twice or three times (n = 24). Eight animals, all adult males, were sighted ten times or more. Fitting a Jolly-Seber model^15^ to individual mark-recapture data (Supplementary Information), we estimated the Broek subpopulation size to have fluctuated between 75 and 115 individuals over the study period (Fig. 1), showing a seasonal pattern consistent with the breeding season of fire salamanders (juveniles emerging between August and October). The mean estimated weekly survival was 0.991 (95% CRI: 0.989–0.993, corresponding to a mean yearly survival of 0.625) and recapture probability was relatively low (mean probability throughout the year 0.12, 95% CRI: 0.11–0.13). In the Broek subpopulation, we collected a total of 207 skin swabs, all from fire salamanders (2013: 57 swabs; 2014: 43 swabs; 2015: 29 swabs and 2016: 78 swabs), none of which tested positive for Bsal. One alpine newt was sighted in 2015 at the Broek site, but not sampled.

In the Bunderbos subpopulation, we sighted 15 and 7 adult fire salamanders in 2015 and in 2016 respectively (47 and 24 site visits, both diurnal and nocturnal), as well as one dead salamander in 2015. At this site we also observed 66 adult/subadult alpine newts in 2015 and 74 in 2016. All sighted post-metamorphic newts and salamanders were sampled for Bsal. For all data prior to 2015, we refer to Spitzen-van der Sluijs *et al*.^8^. In 2015, three fire salamanders out of 16 and two alpine newts out of 66 tested positive for Bsal. Two living salamanders showed loads of 23 and 90 GE (Genomic Equivalent) per swab, one dead salamander showed histopathological lesions and had a GE load of 5.3∙10^3^ per swab. The two alpine newts showed loads of 440 and 322 GE per swab respectively. In 2016, no fire salamanders or alpine newts tested positive for Bsal (0/7 and 0/74 respectively).

**Figure 2:**
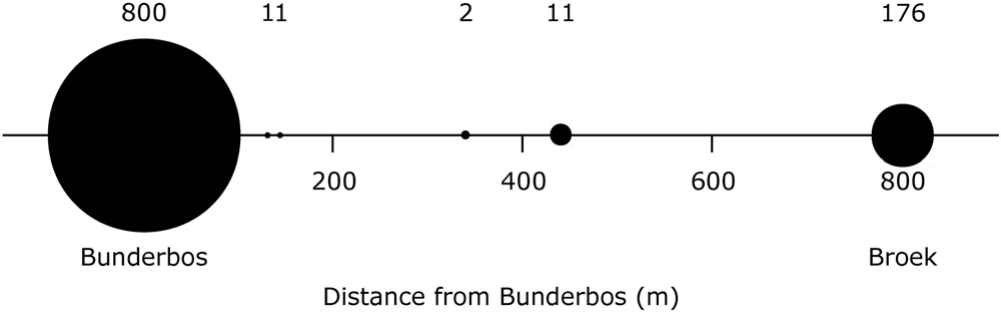
Schematic representation of the distance (in meters) between the Bunderbos and Broek subpopulations and in the matrix in between the two subpopulations. The size of circles corresponds to the number of fire salamanders observed from 2007–2017 (indicated above each circle).

In the intermediate matrix between the two sites, ad hoc sightings of fire salamanders are not exceptional. Between 2010 and 2017, a total of five sightings of larvae, juvenile and adult fire salamanders have been reported from four points which are located between the Bunderbos and Broek (Fig. 2).

### Salamanders from Bunderbos and Broek form a genetic cluster

**Figure 3:**
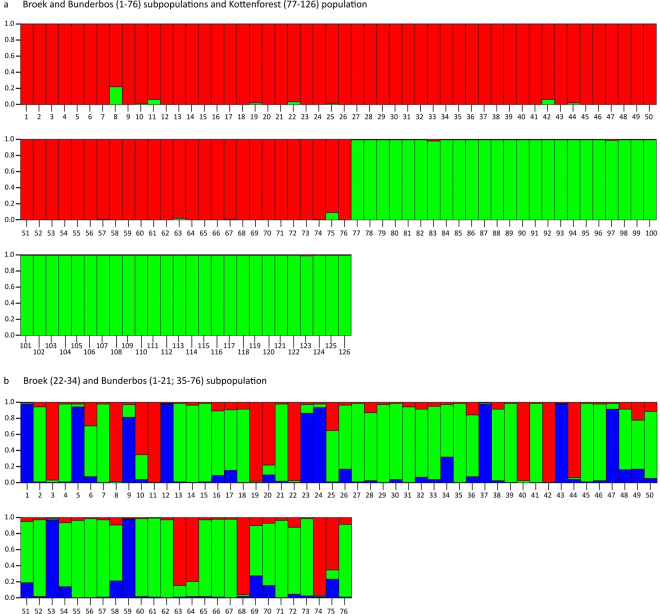
Output from Structure where the most likely number of K is plotted with the data. When K = 2 (red and green), the samples analysed originated from the Broek subpopulation, the Bunderbos subpopulation (1–76) and the Kottenforst population (77–126) (**a**). When K = 3 (red, green and blue), the samples analysed originated from the Broek (22–34) and the Bunderbos subpopulation (1–21, 35–76) only (**b**).

A total of 76 individuals were genotyped for 18 microsatellites: buccal swabs were collected from 63 salamanders originating from the Bunderbos (now in an *ex-situ* conservation program), and from 11 individuals of the Broek subpopulation along with two tail clips from traffic victims from a road immediately next to the Broek subpopulation. The analysis with STRUCTURE showed that the two subpopulations from the Netherlands cluster together genetically as one population when compared to 50 individuals of the reference population from the Kottenforst (Germany; K = 2, mean Ln P(K) = −4745, Delta K = 9469) (Fig. 3a, Supplementary Information Table S1, Fig. S1a,b) while an analysis with the Bunderbos and Broek subpopulations alone did not show a clear differentiation between the two subpopulations (K = 3, Delta K = 37; K = 4, mean Ln P(K) = −2472) (Fig. 3b, Supplementary Information Table S1, Fig. S2a,b). The self-assignment test confirmed the initial result, assigning 100% of the German Kottenforst fire salamanders to that population while individuals from the two Dutch populations were assigned to either one of them (Bunderbos: 63% correctly assigned, 37% assigned to Broek; Broek: 23% correctly assigned, 77% assigned to Bunderbos).

### Physical barriers prevent Bsal transmission

**Figure 4:**
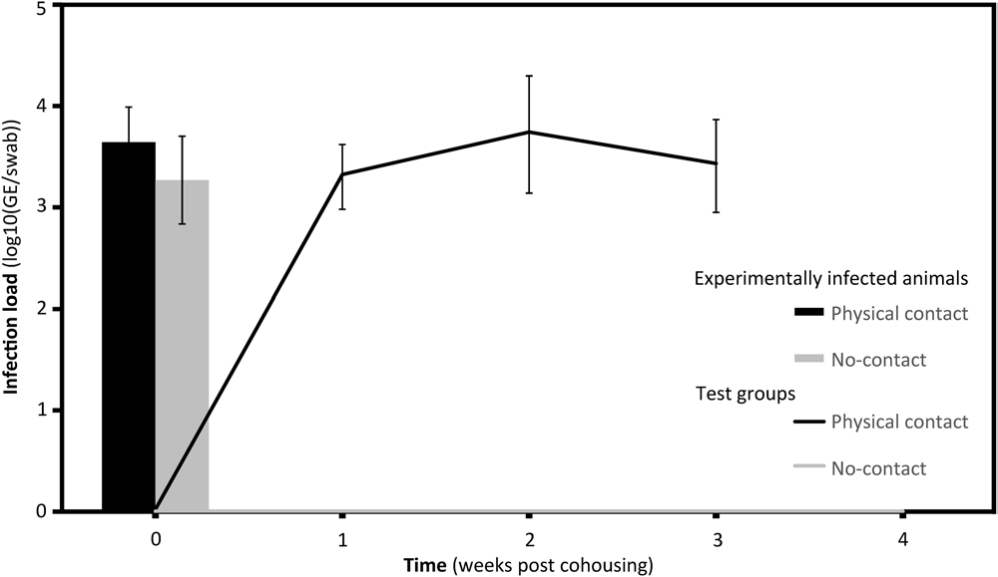
*In vivo* infection experiment with alpine newts (*Ichthyosaura alpestris*). Average infection load for infected newts in each group (Contact vs no-contact group). In the group where physical contact was possible (black), 5 out of 7 newts developed chytridiomycosis while none of the newts in the group where contact was prevented (grey) tested positive for Bsal nor developed chytridiomycosis. Bars: experimentally infected newts in the physical contact group (black) and in the no-contact group (grey). Lines: average infection load of newts that developed clinical signs of chytridiomycosis from the physical contact group (black) and the no-contact group (grey). Error bars represent standard error of the mean.

We conducted a laboratory experiment with alpine newts (*Ichthyosaura alpestris*) to test if Bsal can spread autonomously over short distances. Fourteen pairs, each co-housing one experimentally infected and one non-infected newt, were divided in two treatment groups of seven pairs each: in the first group, infected and non-infected newts were physically separated from each other by a double-sided mesh (mesh size: 1.3 × 1.6 mm.) while in the second group the newts in each pair were free to come into contact with one another. The average infection load of the 14 infected newts before they were co-housed with uninfected animals was 3.5 ± 1.02 (log10 GE/swab; 3.6 ± 0.92 in the contact group and 3.3 ± 1.15 in the no-contact group). One week after co-housing, the infection load was not statistically different compared to the beginning (Mann-Whitney U-test: P = 0.344). Animals were selected randomly for each pairing, as confirmed by the lack of statistically significant differences in loads between the two groups (Mann-Whitney U test; P = 0.535; Fig. 4). At the end of the experiment, after four weeks, the difference in the proportion of individuals infected per group was evident (one-sided Fisher’s exact test, P = 0.01). In the no-contact group, 5/7 of the ‘non-infected’ individuals had become infected; in the no-contact group, none of the non-infected individuals (0/7) tested positive for Bsal.

## Discussion

We found no evidence of Bsal spread between two neighbouring fire salamander subpopulations, despite several possible pathways of dispersion via infected hosts or (a)biotic vectors. The size and trend of the Broek subpopulation suggest that the fungus has so far been unable to bridge the 800-m distance between the two sites. This situation sharply contrasts with available knowledge about Bd^12^ and previous hypotheses about the imminent threat of a rapid Bsal spread^13,14^. The Broek subpopulation has so far persisted in a Bsal-refuge in the vicinity of the Bsal index site, although the exact reason remains unclear. In the remainder of this discussion, we consider the possible scenarios and the implications for future Bsal mitigation.

Autonomous dispersal by Bsal can be ruled out with some certainty. In our experiment, the fungus was unable to cross even the small distance (<1 cm) between the two sides of a permeable (to the pathogen although not to the host) physical barrier in a terrarium. Of non-autonomous dispersal pathways, direct spread of Bsal by infected hosts is arguably the most intuitive. In the surroundings of a known Bsal outbreak site near Liège in Belgium^8^, Bsal-free fire salamanders can still be found as close as 3 km from where the outbreak was originally identified (Stegen, unpublished results). Here, several highways intersecting the continuous forest habitat may represent physical barriers for migrating fire salamanders and other amphibian and non-amphibian vectors. The situation in our study is less clear. Although the matrix between the Broek and Bunderbos sites largely consists of human-modified landscape and small, fragmented habitat patches, we found evidence of at least some connection between the two salamander subpopulations. The matrix allows for overland migration of fire salamanders and alpine newts, and individuals have occasionally been sighted in this connecting landscape (Fig. 2). We found that the Bunderbos and Broek subpopulations cluster together genetically on the basis of microsatellite loci differentiation when compared to the fire salamanders from the Kottenforst (Germany), suggesting they have a shared population history, which is also underpinned by the analysis of mitochondrial D-loop haplotypes (Suppl. Inf. Table S2a, b). Additionally, there is an indication of recent or ongoing gene flow between the two subpopulations (but see Suppl. Inf. for a more detailed discussion of the genetic analysis). More in general, both healthy fire salamanders and alpine newts have sufficient dispersal capabilities to cover the distance between the two sites^16,17^. However, the dispersal capability of Bsal-infected individuals is a more relevant parameter here. Canessa *et al*.^11^ predict that infected fire salamanders would move on average less than 100 m before succumbing to infection. Schmidt *et al*.^13^, while predicting a rapid spatial spread of Bsal, also recognise the possibility that infected individuals may not move far enough to transmit the disease to neighbouring forest patches.

Ultimately, spread is a stochastic process, and a larger source host population will produce a greater number of dispersers, particularly rare long-range ones. In this sense, dispersal of Bsal from the original site in the Bunderbos to the Broek subpopulation may have been impeded by the rapid collapse of the fire salamander hosts at the former site. The virulence of the pathogen may have hindered its spread by functionally removing most potential dispersers, and thus further reducing the stochastic chances. On the other hand, alpine newts persist in the Bunderbos at higher densities than fire salamanders. Newts may survive Bsal infection and even carry it asymptomatically^10^ and are known to have higher dispersal abilities than fire salamanders^18–20^, making them potentially more important dispersers of the pathogen. However, no infected newts were found in the Bunderbos in 2016, and only one newt was sighted in the newly discovered fire salamander subpopulation, suggesting this host also provides low chances for Bsal dispersal, at least in this area.

If dispersal by infected hosts is restricted, whether by dispersal abilities of the hosts themselves, by sub-optimal matrix permeability, or by the small number of available hosts (possibly as a result of Bsal epizootic dynamics at the source), vectors may represent the next most likely pathways. Dispersal by biotic (non-susceptible) vectors is possible: Stegen *et al*.^10^ demonstrated Bsal spores can attach themselves to scales of goose feet. Bird vectors are also unlikely to be significantly affected by sub-optimal permeability of the matrix between the two sites.

As for abiotic vectors, waterways are considered highly suitable for fungal survival and spread^10^: a stream directly connects the two subpopulations in our study. More than half of this stream is subterranean, and the aboveground part contains fish, possibly making it unsuitable habitat for vector species such as alpine newts. Fire salamanders in the Bunderbos have been demonstrated to deposit their larvae upstream: zoospores and fire salamander larvae can be expected to flush to the downstream naive subpopulation. However, the current absence of Bsal from the Broek subpopulation suggests to date spread by water or by such passive vectors as flushed amphibian larvae has also been unsuccessful, whether as a result of a deterministic (e.g. due to barriers preventing vector movements) or stochastic process (e.g. due to low numbers of potential vectors and consequent low chances of successful dispersal).

Our results provide important information about the potential of Bsal to disperse rapidly through the landscape, suggesting such potential might not be as high as previously thought^13,14^ or as its congeneric species Bd^12^. In turn, this information has important implications for Bsal mitigation. Although mitigation is likely to prove highly challenging during the epizootic event^11^, if the risk of spread remains low the disease might effectively eradicate itself by extirpating its hosts; mitigation actions could be implemented during or after the outbreak to further reduce spread (for example by actively removing individuals^11^). Population reinforcement and reintroductions might be implemented after the disease has faded out, or to buffer remaining populations against stochastic extinctions.

Moreover, the possibility that Bsal is indeed a weaker disperser than originally hypothesized further reinforces the need to prevent its human-mediated dispersal. The currently known distribution of Bsal in Europe is discontinuous, with apparent jumps^8^ for which human-mediated dispersal cannot be ruled out under current evidence. Quarantine and biosafety protocols should be rigorously implemented, and more radical actions considered (such as restriction of access by quarantine fences). The case we have described may provide directions for disease management in highly threatened, range-restricted, isolated or locally endemic salamander species, such as *Salamandra atra pasubiensis*, *S*. *atra aurorae*, *S*. *lanzai* or *Calotriton arnoldi*, which might face fast extinction in the event of Bsal arrival within their ranges.

## Methods

### Site

We do not disclose the exact location of the novel site (Broek subpopulation) to prevent pathogen pollution or otherwise harmful activities^21,22^. The new site is small (0.57 ha.), is located within a one km radius of the Bunderbos^7^ and consists of an artificial habitat: a fast-flowing stream with a steep, concreted slope passes through the area, which is void of a water body suitable for fire salamander reproduction. Both the terrestrial and aquatic habitat are marginal. Multiple creeks merge underground into this stream, including water that originates from the Bunderbos area, which was the first location at which Bsal was detected^7^. Old maps of the area, dating back to 1868, show a natural connection of the current stream, through meadow and brook land forest with the Bunderbos. The landscape between the two subpopulations is characterized by an urbanized and agricultural zone. We checked the national databank flora and fauna for sightings of fire salamanders in this matrix in the period 2007–2017 (www.ndff-ecogrid.nl; accessed 16 Nov. 2017). Elevation of the Broek subpopulation ranges between 40 m and 56 m above sea level (www.ahn.nl; accessed 7 Sept. 2017), and the vegetation consists of poplar trees, shrubs, bushes and grassland.

### Inferring demographics of the new fire salamander population (Broek subpopulation)

Standardized monitoring of the fire salamanders started immediately upon discovery of the Broek subpopulation in October 2013. Transect counts were continuously done after sunset, either in the late evening or at night, under humid or wet conditions with temperatures ≥ 5 °C, according to the national standard to monitor fire salamanders^23^. The transect covers the entire area and measures 665 m in total added length. Over the period October 2013 – October 2016, the site was visited 64 times: 8 times in 2013 (October – December); 22 times in 2014 (April, May, August – December); 24 times in 2015 (January – December); and 10 times in 2016 (January - October). The mean interval between site visits was 17.6 days (range: 1–122; median: 61.5).

During all 64 visits between October 9^th^ 2013 and October 20^th^ 2016, the dorsal pattern of each individual fire salamander was recorded by photography. These patterns, unique for each individual^24^, allowed us to identify recaptures on the basis of dorsal spot patterns using the program AMPHIDENT^25^. We used these mark-recapture data to estimate the survival, recapture probability and population size using the Jolly-Seber open-population model^15^. We assumed constant apparent survival, and modelled the probability of entry and that of recapture using a cosine function to reflect seasonal variation in salamander migration (entry) and activity patterns (detection). We rescaled survival and entry on a weekly period, to account for the variable intervals between surveys. We fitted the model in JAGS^15,26^ using uninformative priors for all parameters (model code in Supplementary Information). We drew 50,000 samples from the posterior distributions of all parameters, from three Markov chains with overdispersed initial values, after discarding the first 25,000 as a burn-in and applying a thinning rate of 10. We assessed convergence by visual inspection of the chain histories, and through the R-hat statistic.

### Detection of Bsal

Ventral skin swabs were taken from post-metamorphic salamanders and newts, using aluminium sterile cotton-tipped dryswabs (rayon-dacron, COPAN, UNSPSC CODE 41104116) following the procedure and biosecurity measures described in Hyatt *et al*.^27^ and Van Rooij *et al*.^28^. All samples were kept frozen at −20 °C until further analysis for the presence of Bsal DNA through real-time PCR, as described by Blooi *et al*.^29^. Skin histopathology as described in Martel *et al*.^6^ was performed to detect Bsal infection on dead salamanders.

### Genetic analyses

We collected genetic samples - buccal swabs - from fire salamanders at the Broek and the Bunderbos subpopulation to test the origin of the Broek subpopulation and to draw conclusions on the overall genetic constitution of both subpopulations. We hypothesize that the Broek subpopulation has a relict origin, although an anthropogenic introduction has been suggested and is also deemed possible. The samples were used to assess the population structure between the Broek and the Bunderbos subpopulations on the basis of neutrally evolving microsatellite loci. Therefore, samples from the Broek and the Bunderbos subpopulation were genotyped for 18 microsatellite loci as described in Steinfartz *et al*.^30^ and Hendrix *et al*.^31^ and compared to the well-studied population of fire salamanders in the Kottenforst, near Bonn (north-Rhine Westphalia in Germany, approximately 100 km from the Bunderbos as the crow flies)^17,32,33^. We assessed recent gene flow between the Broek and the Bunderbos subpopulation and the Kottenforst (Germany) using the program STRUCTURE, followed by Structure Harvester to identify the most probable number of populations (K)^34–38^. Structure was run using 20 iterations for each K and K was *a priori* assumed to be between 1 and 20 clusters, each iteration had a burn-in of 100.000 runs followed by 2.000.000 runs after burn-in. No *a priori* information on sample origin (LOCPRIOR) was fed into the program. The most probably K was defined using the delta K method as described by Evanno *et al*.^39^ as well as the posterior likelihood of the data as described by Pritchard *et al*.^34^, only when both methods identified the same K, we inferred this K as the most likely. Lastly, we used the program GENECLASS2^40^ in order to assign individuals to their respective subpopulation (Bunderbos and Broek, e.g. Valbuena-Ureña *et al*.^41^).

### Physical barriers act to restrain Bsal transmission

Although soil can act as a vector for Bsal^10^, it is unknown whether the fungus can spread actively over short distances. We studied the role of physical barriers in *Bsal* transmission between infected and non-infected alpine newts (*Ichthyosaura alpestris*). Alpine newts were chosen because they act as vector but can survive infection and co-occur with *Salamandra* populations frequently. During the experiment, direct physical contact between infected and non-infected individuals was either allowed (contact group) or prevented (no-contact group) by placing a physical barrier (double sided mesh; mesh size: 1.3 mm × 1.6 mm, the sides placed 0.5 cm apart) in the middle of the containers from the no-contact group. At the onset of the experiment, 14 newts were infected with 10^5^ zoospores of the Bsal type strain (AMFP13/1) suspended in 1 ml distilled water following Martel *et al*.^42^ to ensure 100% *Bsal* prevalence. During the experiment, all animals were clinically examined every day. Seven days after the initial inoculation we collected skin swabs to determine the infection status and load for each animal. Each infected individual was randomly assigned to either the contact or no-contact group and co-housed with a non-infected individual. Each group (contact versus no-contact) therefore contained seven pairs. After seven days of co-housing, the 14 experimentally infected individuals were swabbed, removed from the experiment and heat treated to cure the animal from the Bsal infection as described by Blooi *et al*.^43^. Hereafter, skin swabs were collected every seven days for three consecutive weeks from the non-infected animals to determine the *Bsal* infection loads. An animal was considered infected when two consecutive swabs were positive, or if the genomic load was higher than 3 log10 (GE; genomic equivalents). As soon as a newt tested positive for Bsal, the animal was removed from the experiment and treated as described by Blooi *et al*. (2015). Each container (19 × 12 × 7 cm) was filled with a layer of unsterilized and moisturized forest soil – from a Bsal-free forest–, and kept constantly at 15 °C. Crickets, which were also unable to cross the barrier, were provided as food items *ad libitum* twice a week. To avoid cross contamination, each individual was handled with a new pair of nitrile gloves. Prior to taking part in the experiment all animals were tested for, and proved free of Bsal, Bd and ranavirus – two other infections causing major amphibian diseases.

### Ethics statement

All methods involving animals were approved by and carried out in accordance with the guidelines and regulations of permit EC2015/29 issued by the ethical committee of Ghent University and permit FF/75 A/2016/015 issued by the Netherlands Enterprise Agency.

## Electronic supplementary material

Supplementary information
