# Supplementary material for: Post-epizootic salamander persistence in a disease-free refugium suggests poor dispersal ability of Batrachochytrium salamandrivorans
Source: Sci Rep. 2018 Feb 28;8:3800. doi: 10.1038/s41598-018-22225-9 (PMC5830533; doi:10.1038/s41598-018-22225-9)
Supplement: Supplementary file 1 — Supplementary information [file 41598_2018_22225_MOESM1_ESM.pdf]

Supplemental Information

Post-epizootic salamander persistence in a disease-free refugium suggests poor dispersal ability of *Batrachochytrium salamandrivorans*

Annemarieke Spitzen, Gwij Stegen, Sergé Bogaerts, Stefano Canessa, Sebastian Steinfartz, Nico Janssen, Wilbert Bosman, Frank Pasmans and An Martel. *Scientific Reports* (2018).

Supplementary information that accompanies this paper:

More detailed methods, results and discussion section on the analysis of genetic analyses **3**

Supplemental Information Table S1. Evanno table identifying the most probable hypothetical K within the dataset. **8**

Supplemental Information Table S2a. Mitochondrial D-loop analysis. **9**
Supplemental Information Table S2b. Comparison of the nucleotide sequences **9**

Supplementary Information Table S3. Genetic diversity indices for Bunderbos **10**

Supplementary information Figure S1. Selection of the most likely number of K **11**

Supplementary information Figure S2. Selection of the most likely number of K **13**

JAGS code for the Jolly-Seber model for estimation of *S. salamandra* population size. **15**

References **17**

More detailed methods, results and discussion section on the analysis of genetic analyses

Methods

The samples were used to determine the origin of the Dutch fire salamanders by analysing the mitochondrial D-loop and to assess the population structure between the Broek and the Bunderbos subpopulations on the basis of neutrally evolving microsatellite loci. Samples from two focal populations were sequenced for the mitochondrial D-loop using primers as described in Steinfartz *et al*. (2000). Obtained sequences were categorized according to D-loop haplotypes found across Europe following the study of Weitere *et al*. (2004) to determine whether the populations consist of native fire salamanders or whether they might have been introduced in the past from other regions in Europe. In addition, samples from the Broek and the Bunderbos subpopulation were genotyped for 18 microsatellite loci as described in Steinfartz *et al*. (2004) and Hendrix *et al*. (2010) and compared to the well-studied population of fire salamanders in the Kottenforst, near Bonn (north-Rhine Westphalia in Germany, approximately 100 km from the Bunderbos as the crow flies) (Hendrix et al. 2017, Steinfartz et al. 2007, Caspers et al. 2014). This population serves here as a reference population in terms of genetic composition. Population genetic analyses were performed with programs GENALEX 6.5b2 (Peakell & Smouse 2012) and ARLEQUIN ver3.5.1.2. (Excoffier & Lischer 2010) to determine deviations from Hardy–Weinberg equilibrium in the Bunderbos subpopulation for each locus, which provides an exact probability value (Guo & Thompson, 1992). We then estimated the populations' inbreeding coefficients (F_{IS} and F_{ST}).

Results

Haplotype

For the mitochondrial D-loop analysis, 31 individual samples were analysed (20 from Bunderbos and 11 from Broek). Eight of these were identified as Type Ia (western evolutionary lineage) and one as a Type II (eastern evolutionary lineage) on the basis of a major haplotype network which spans Europe (Weitere et al. 2004) (Supplementary Table S2a), which have also recolonized major parts of western Germany while the remaining 22 samples represent a new D-loop haplotype, that differed by a single mutational step from Type I (Supplementary Table 2b).

Genetic diversity

The genetic diversity of the Dutch fire salamanders, which was assessed using the samples from Bunderbos, indicated that 5 loci deviated from Hardy-Weinberg equilibrium (Supplementary Table 3), while only two loci did so for the reference population from the Kottenforst. Across the 18 genotyped loci of individuals from Bunderbos, we detected a total of 87 alleles with a mean number of 4.83 alleles per locus. The mean number of effective alleles was 2.40 (range: 1.16-3.88). Mean observed heterozygosity (H_o=0.48) was lower than expected (H_e=0.53) and the mean inbreeding coefficient (F_{IS}) was 0.10. Compared to the Kottenforst, 88 alleles with a mean number of 4.89 alleles per locus were detected. The mean number of alleles was 2.65 (range: 1.25-4.57). Mean observed heterozygosity (H_o=0.55) was also lower than expected (H_e=0.57). Mean inbreeding coefficient was (F_{IS}) 0.05. The genetic distance (F_{ST}) between the two populations (Kottenforst and Bunderbos) was estimated to 0.20.

Discussion

Haplotype

We found that the Bunderbos and Broek subpopulations cluster together genetically on the basis of microsatellite loci differentiation when compared to the fire salamanders from the Kottenforst (Germany), suggesting they have a shared population history, which is also underpinned by the analysis of mitochondrial D-loop haplotypes. They most likely have a shared origin with the German Western evolutionary lineage, which has recolonized this part of Central Europe following the last glaciation approximately 9000 years ago (Weitere et al. 2004, Steinfartz et al., 2007). The presence of the Type Ia lineage, in combination with the occurrence of a new D-loop haplotype only a single mutation step apart, makes it very unlikely that the Bunderbos population has been founded on introduced salamanders from other areas in Europe, which debunks a pertinacious rumour in the Netherlands.

Similar genetic constitution of the subpopulations

The outcome of the STRUCTURE analysis indicating the presence of three genetic clusters (K=3; see Supplementary Fig. S2b), has no impact on the observed genetic population structure and differentiation between the two subpopulations. Here, only the individual genotypes of the Bunderbos and Broek subpopulations were analysed and the clustering of individuals does not follow a structure of underlying subpopulations. It is difficult and speculative to say why we observe a K=3 at this level. Possibly, this might be because the samples from Bunderbos already represent non-random samples as they were collected after the collapse of the subpopulation due to Bsal. Such as biased sampling could violate the assumptions of Hardy-Weinberg equilibrium. Most importantly, however, we do not see

that the estimation of K=3 has an impact on the population structure and genetic differentiation of both subpopulations, stating that the overall genetic constitution of both subpopulations should be rather similar and is not influenced by genetic structure based on microsatellite loci differentiation population structure.

Genetic diversity

Although the microsatellite loci analysis revealed significant deviations from HWE for 5 microsatellite loci, two of these also deviated from HWE in the German reference population. The mean inbreeding coefficient (FIS) was low, which allows us to conclude that there is no significant excess or deficiency of heterozygotes that indicates a recent population bottleneck followed by inbreeding (non-random mating) or genetic drift. Alternative explanations for the deviation from HWE might be the violation of the assumptions of the theorem (e.g. finite population, non-random mating, mutation, immigration) or the presence of null alleles within the sample. Immigration and non-random mating can likely be discarded as causative agents of the deviation as there is no substantial other fire salamander population from which fire salamanders might have immigrated to the Bunderbos population, and fire salamanders are polygynandrous (polyandrous females in Caspers et al. (2014); polygynous males in Helfer et al. (2012)). This assumption is corroborated by recent empirical studies on the critically endangered Montseny brook newt (*Calotriton arnoldi* (Valbuena-Ureña et al. (2017)) and urban fire salamanders in Spain (Lourenço et al., 2017). These populations show no signs of inbreeding or loss of heterozygosity despite facing strong habitat fragmentation and display low census population sizes. Possibly, the loci C2 and C3 act as null alleles because the allele frequencies are reduced, but did not show a significant statistical bias or a shifted genetic variation when

compared with the reference population. A third reason for the deviation from HWE might be the timing of collection of the samples from Bunderbos, i.e. after the population collapse. Therefore, our sampling cannot be considered random and the deviations from HWE might be the result of an unknown underlying process. Still, it seems that there is sufficient genetic variability left among the surviving individuals, which should suffice in maintaining an ex-situ breeding program for a possible re-introduction program in the future.

Supplemental Information Table S1. Evanno table identifying the most probable hypothetical K within the dataset. #K: number of hypothetical populations in run, Reps: number of replications performed per K, Mean LnP(K): Mean of the log likelihood of the data, Stdev Ln P(K): standard deviation log likelihood of the data, Ln'(K): first order rate of change in the likelihood of the data, |L''(K)|: absolute value for the second order rate of change in the likelihood of the data, Delta K: *ad hoc* quantity related to the second order rate of change of the log probability of the data between successive K values.

	# K	Reps	Mean LnP(K)	Stdev Ln P(K)	Ln'(K)	|Ln''(K)|	Delta K
Broek + Bunderbos + Kottenforst samples	1	20	-5710	0,3	NA	NA	NA
	2	20	-4745	0,1	965	863	9469
	3	20	-4644	64	101	19	0,3
	4	20	-4562	12	82	39	3
	5	20	-4519	12	43	NA	NA
Broek + Bunderbos samples	1	20	-2679	0,3	NA	NA	NA
	2	20	-2612	25	67	34	13
	3	20	-2512	16	100	60	37
	4	20	-2472	49	40	156	32
	5	20	-2588	187	-116	NA	NA

Supplemental Information Table S2a. Mitochondrial D-loop analysis. Classification of haplotypes found in the Bunderbos and the Broek fire salamander subpopulations compared to the Kottenforst data (Weitere et al. (2004)).

Locality	No. of individuals analysed for the D-loop	Haplotypes assignment and frequencies
Bunderbos	20	Type Ia (35%), Type I-like (65%)
Broek	11	Type Ia (9%), Type I-like (82%), Type II (9%)
Kottenforst	37	Type Ia: 78%, Type Ib (22%)

Supplemental Information Table S2b. Comparison of the nucleotide sequences in the mitochondrial D-loop haplotypes used in this study. All sequences where previously known (Weitere et al. 2004), except for the type I-like sequence.

D-loop haplotype	Nucleotide site										
	1-26	27	28-274	275	276-336	337	338-428	429	430-589	590	591-724
Type I	IDENTICAL	C	IDENTICAL	A	IDENTICAL	-	IDENTICAL	A	IDENTICAL	C	IDENTICAL
Type Ia		C		A		-		A		-	
Type Ib		C		A		T		A		C	
Type I-like		C		T		-		A		C	
Type II		T		A		-		A		C	
Type IIa		T		A		-		G		C	

Supplementary Information Table S3. Genetic diversity indices for Bunderbos based on 18 microsatellite loci. Significance testing was corrected for multiple comparisons by the Bonferroni correction (adjusted p-value = 0.0028). N: number of samples, Na: number of alleles, Ne: effective number of alleles, I: Shannon's information index, Ho: observed heterozygosity, He: expected heterozygosity, χ^2: Chi-square test for Hardy-Weinberg equilibrium, Signif: p-value for χ^2 test, Post Bonferroni corr: significance of p-value after Bonferroni correction (ns: not significant; SIGNIF: significant), FIS: inbreeding coefficient

	Locus	N	Na	Ne	I	Ho	He	χ^2	Significance	Post Bonferroni corr*	F_{IS}
BUNDERBOS	**SalE8**	60	5	3,36	1,32	0,75	0,7	11,25	0,338	ns	-0,06
	**IIA6**	59	6	3,88	1,49	0,76	0,74	43,81	0	**SIGNIF**	-0,02
	**E11**	61	7	2,76	1,24	0,61	0,64	42,46	0,004	ns	0,06
	**IA6**	60	5	2,15	1,06	0,45	0,53	41,49	0	**SIGNIF**	0,17
	**B11**	62	8	3,02	1,38	0,73	0,67	13,23	0,992	ns	-0,08
	**SalE6**	62	3	1,35	0,51	0,29	0,26	1,79	0,618	ns	-0,12
	**C3**	62	4	1,16	0,32	0,15	0,14	0,38	0,999	ns	-0,05
	**C2**	53	3	1,4	0,49	0,17	0,28	13,31	0,004	ns	0,41
	**Sal3**	61	5	1,62	0,77	0,39	0,38	24,5	0,006	ns	-0,02
	**SalE7**	62	5	2,64	1,16	0,6	0,62	13,53	0,196	ns	0,05
	**SalE11**	61	4	3,34	1,28	0,69	0,7	32,9	0	**SIGNIF**	0,03
	**SalE14**	61	3	1,93	0,73	0,39	0,48	3,68	0,299	ns	0,19
	**F10**	52	5	2,13	1	0,15	0,53	78,34	0	**SIGNIF**	0,71
	**SalE2**	61	7	3,18	1,38	0,23	0,69	212,21	0	**SIGNIF**	0,67
	**SalE12**	61	6	3,24	1,33	0,69	0,69	13,9	0,533	ns	0,01
	**G9**	63	4	2,17	0,99	0,57	0,54	9,43	0,151	ns	-0,05
	**SalE5**	63	3	2,1	0,87	0,57	0,52	1	0,801	ns	-0,08
	**Sal29**	62	4	1,74	0,74	0,47	0,43	11,33	0,079	ns	-0,09
	**MEAN**	60,33	4,83	2,4	1	0,48	0,53	-	-	-	0,05
	**SE**	0,71	0,35	0,19	0,08	0,05	0,04	-	-	-	0,06

* adjusted p-value: 0,0028

Supplementary information Figure S1. Selection of the most likely number of K in the microsatellite data from the Bunderbos, Broek and Kottenforst fire salamanders. Plots illustrate the selection of the most likely number of clusters by the two most common two methods: The Ln P(K) method (**A**) and the delta K method (**B**). The first method identifies the most likely K when the likelihood of the data at a specific K-value is maximal or, if there is no clear maximum, when the likelihood reaches a plateau. The second method identifies the most likely K when the second order rate of change in the likelihood of the data between successive values for K is largest. In both cases, the most likely K equal 2.

A

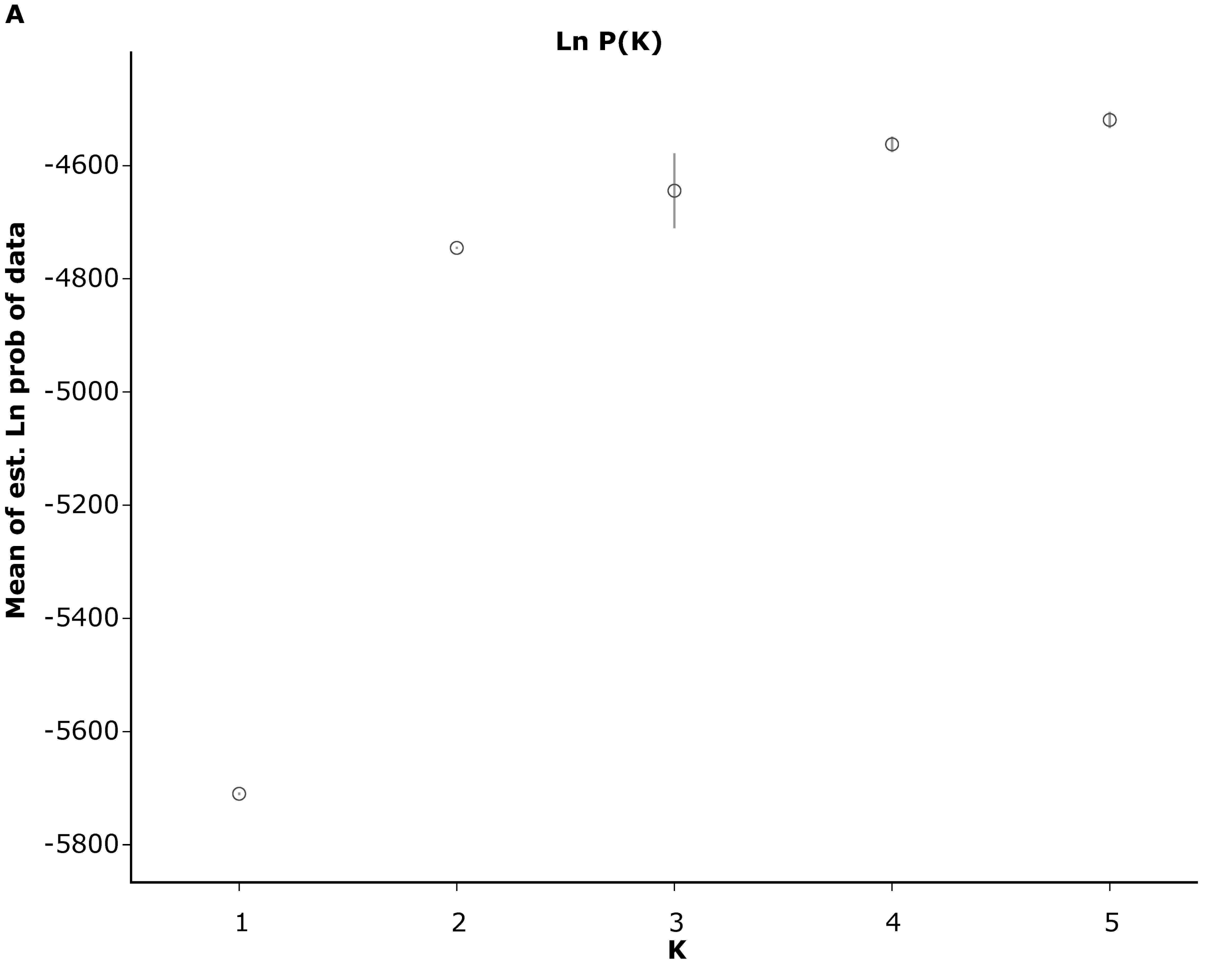

B

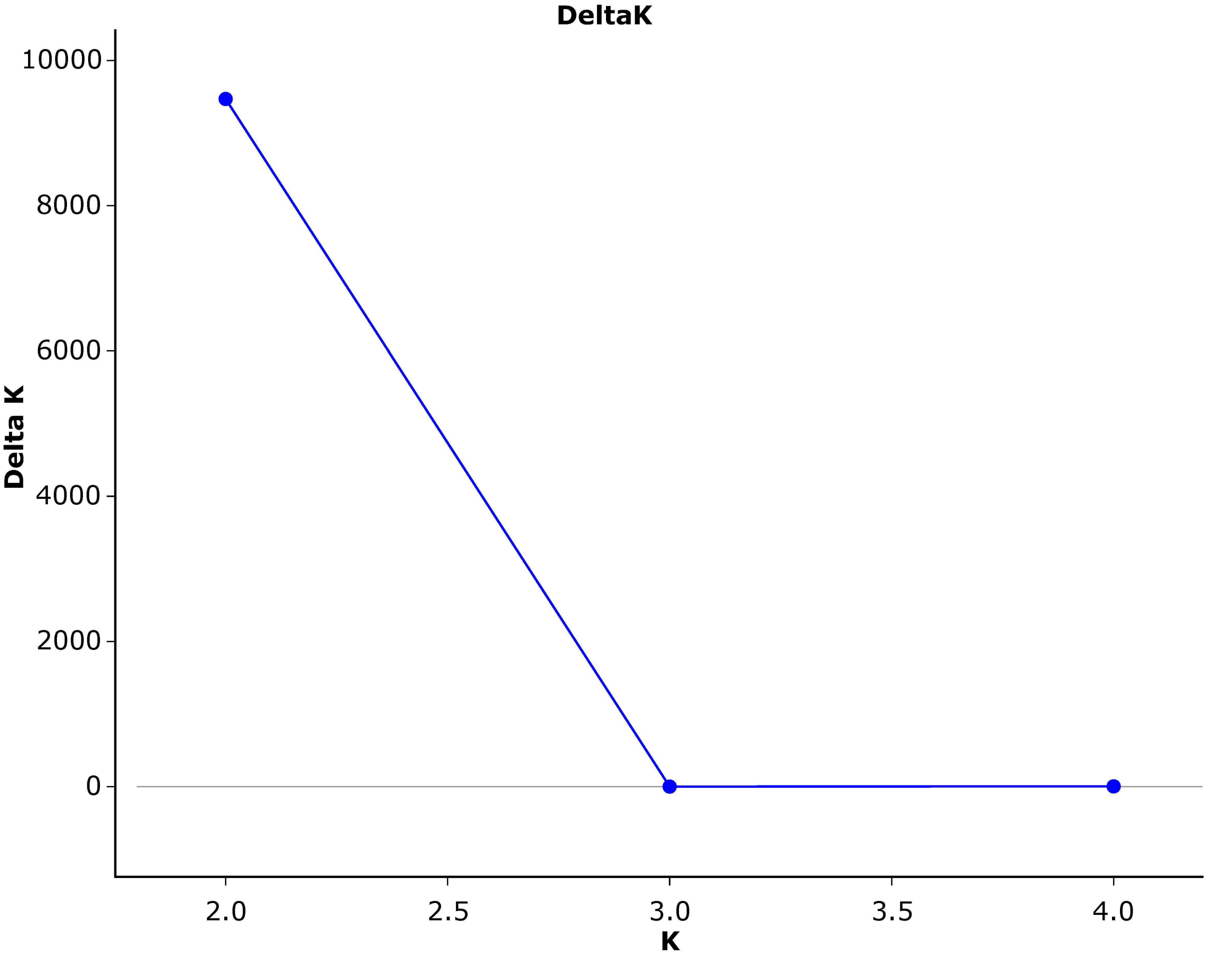

Supplementary information Figure S2. Selection of the most likely number of K in the microsatellite data from the Bunderbos and Broek fire salamander subpopulations. Plots illustrate the selection of the most likely number of clusters by the two most common two methods: The Ln P(K) method (**A**) and the delta K method (**B**). The first method identifies the most likely K when the likelihood of the data at a specific K-value is maximal or, if there is no clear maximum, when the likelihood reaches a plateau. The second method identifies the most likely K when the second order rate of change in the likelihood of the data between successive values for K is largest. Using the Ln P(K) method, the most likely K is 4, while the delta K method identifies K=3 as the most likely K.

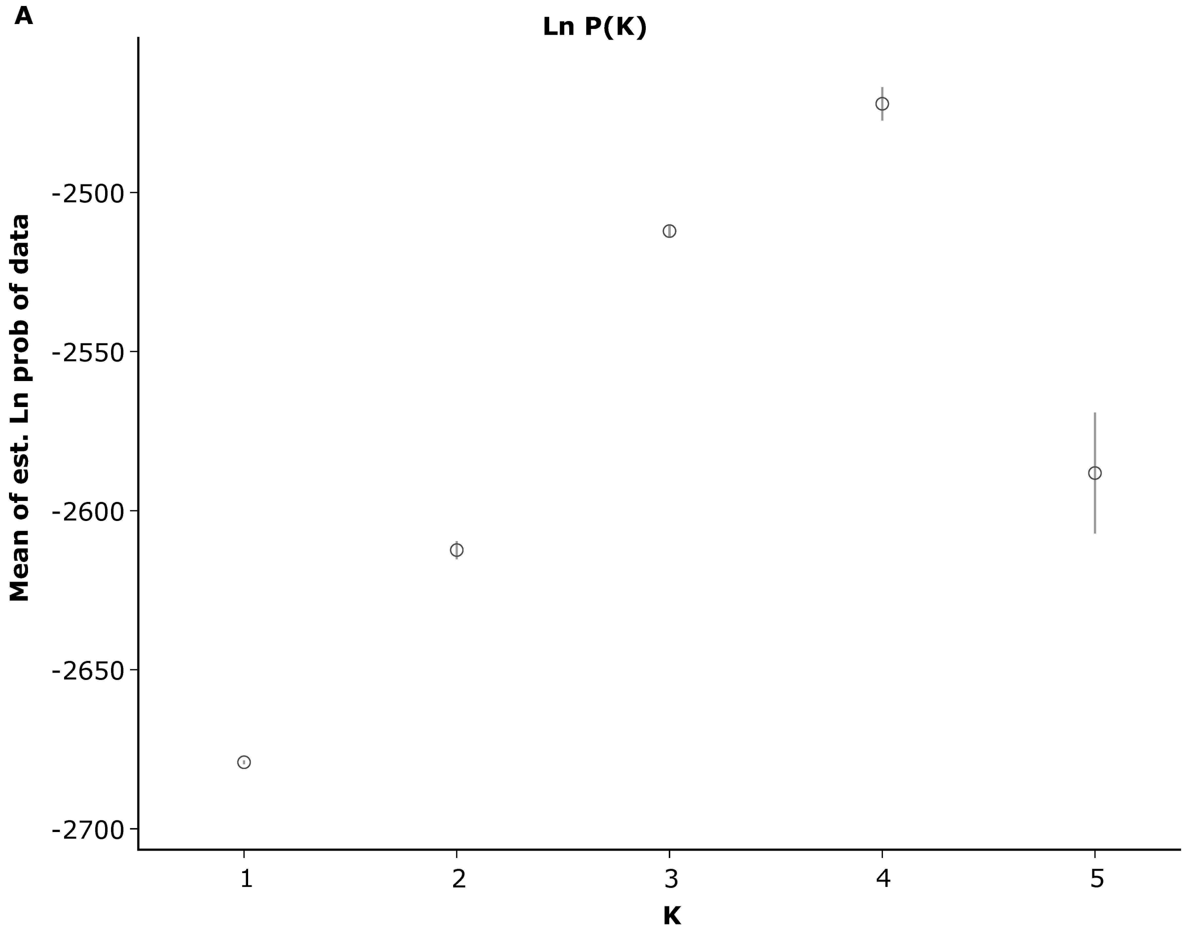

B

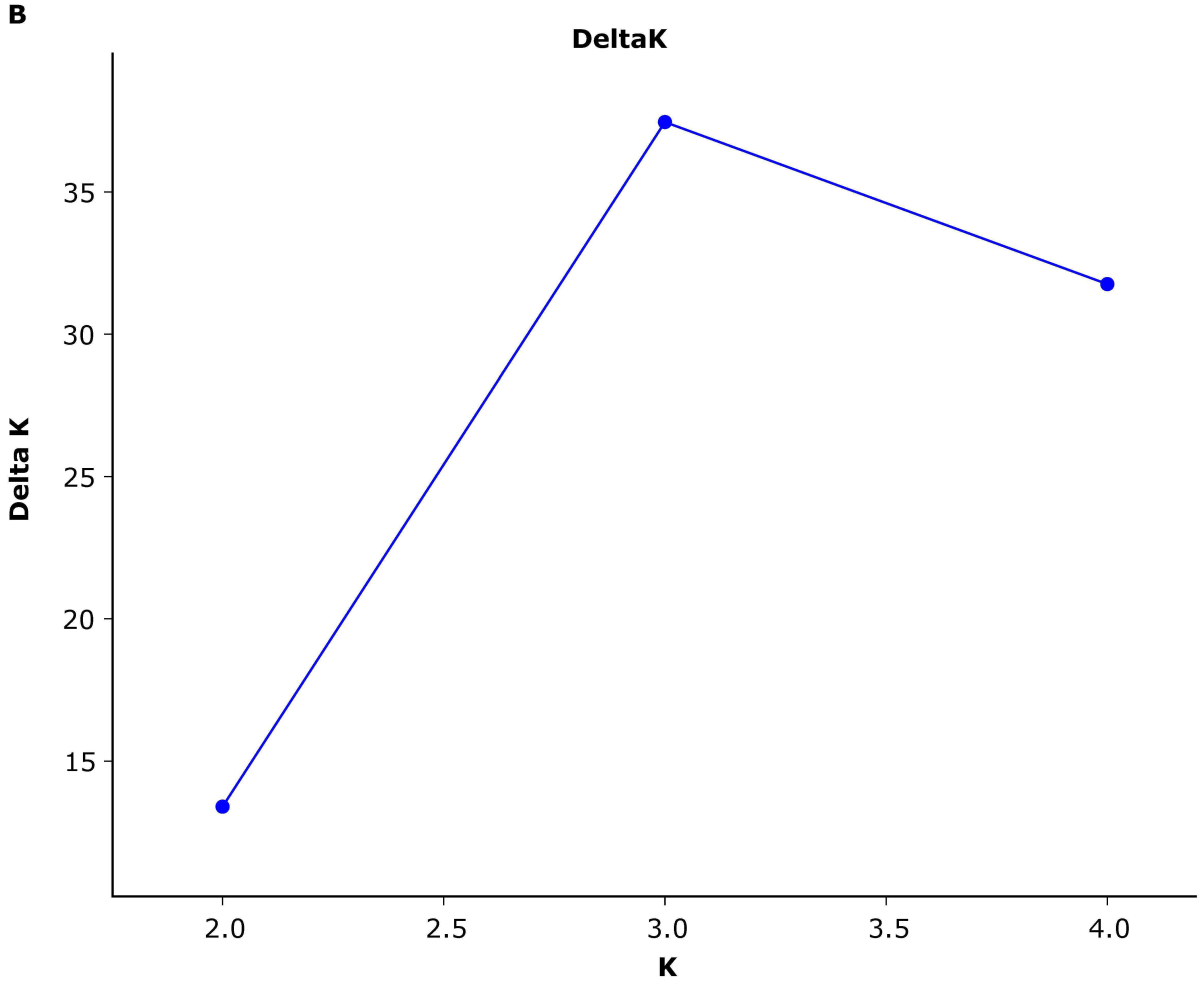

JAGS code for the Jolly-Seber model for estimation of *S. salamandra* population size. Model adapted from Ch. 10 in Kéry & Schaub (2011)

```
MODEL{
# ===========================================================================
# LIKELIHOOD
	for(i in 1:M){
# The latent state must be 1 in the first occasion
		z[i,1] <- 1for(t in 2:n.surv){
# State
		z[i,t] ~ dcat(ps[z[i,t-1],t-1,])
# Observation
		y[i,t] ~ dcat(po[z[i,t],t-1,])
		}
	}
# ===========================================================================
# PRIORS AND CONSTRAINTS
# Phi: survival rate (constant)
	for(t in 1:(n.surv-1)){
		phi[t] <- pow(mean.phi,days[t]/7) # Weekly survival
# Gamma: entry probability - varies seasonally
		gamma[t] <- pow(logit_gamma[t],days[t]/7)
		# Weekly entry (to reflect variable length of intervals between surveys)
		logit(logit_gamma[t]) <- a.g+b.g*cos(date[t]*2*3.1416/365-offset.g)
# P: detection probability - varies seasonally
		logit(p[t]) <- a.p+b.p*cos(date[t]*2*3.1416/365-offset.p)
	}
# Transition matrices
# Define probabilities of state S(t+1) given S(t)
	for(t in 1:(n.surv-1)){
		ps[1,t,1] <- 1-gamma[t]
		ps[1,t,2] <- gamma[t]
		ps[1,t,3] <- 0
		ps[2,t,1] <- 0
		ps[2,t,2] <- phi[t]
		ps[2,t,3] <- 1-phi[t]
		ps[3,t,1] <- 0
		ps[3,t,2] <- 0
		ps[3,t,3] <- 1
# Define probabilities of O(t) given S(t)
		po[1,t,1] <- 0
		po[1,t,2] <- 1
		po[2,t,1] <- p[t]
		po[2,t,2] <- 1-p[t]
		po[3,t,1] <- 0
		po[3,t,2] <- 1
	}
```

```
# Priors
mean.phi ~ dunif(0.5,1)
mean.gamma ~ dunif(0,0.2)
a.p ~ dnorm(0,0.001)
b.p ~ dnorm(0,0.001)
offset.p ~ dunif(0, 6.283185)
a.g ~ dnorm(0,0.001)
b.g ~ dnorm(0,0.001)
offset.g ~ dunif(0, 6.283185)
# ======================================================================
# POPULATION SIZE
    for(t in 1:(n.surv-1)){
        qgamma[t] <- 1-gamma[t]
    }
cprob[1] <- gamma[1]
    for(t in 2:(n.surv-1)){
        cprob[t] <- gamma[t]*prod(qgamma[1:(t-1)])
    }
psi <- sum(cprob[])
    for(t in 1:(n.surv-1)){
        b[t] <- cprob[t]/psi
    }
# Living individuals at time t-1
    for(i in 1:M){
        for(t in 2:n.surv){
            al[i,t-1] <- equals(z[i,t],2)
        }
        for(t in 1:(n.surv-1)){
            d[i,t] <- equals(z[i,t]-al[i,t],0)
        }
    alive[i] <- sum(al[i,])
    }
    for( t in 1:(n.surv-1)){
        N[t] <- sum(al[,t])
        B[t] <- sum(d[,t])
    } # t
    for(i in 1:M){
        w[i] <- 1-equals(alive[i],0)
    }
# Total size of each population
Nsup <- sum(w[])

}
```

References

Steinfartz, S., Veith, M. & Tautz, D. Mitochondrial sequence analysis of *Salamandra* taxa suggests old splits of major lineages and postglacial recolonizations of Central Europe from distinct source populations of *Salamandra salamandra*. *Mol. Ecol.* **9,** 397–410 (2000).

Weitere, M., Tautz, D., Neumann, D. & Steinfartz, S. Adaptive divergence vs. environmental plasticity: tracing local genetic adaptation of metamorphosis traits in salamanders. *Mol. Ecol.* **13**, 1665–1677 (2004).

Steinfartz, S., Küsters, D. & Tautz, D. Isolation of polymorphic tetranucleotide microsatellite loci in the Fire Salamander *Salamandra salamandra* (Amphibia: Caudata). *Mol. Ecol. Notes* **4,** 626–628 (2004).

Hendrix, R., Hauswaldt, J. S., Veith, M. & Steinfartz, S. Strong correlation between cross-amplification success and genetic distance across all members of 'True Salamanders' (Amphibia: Salamandridae) revealed by *Salamandra salamandra*-specific microsatellite loci. *Mol. Ecol. Resour.* **10,** 1038–1047 (2010).

Hendrix, R., Schmidt, B. R., Schaub, M., Krause, E. T. & Steinfartz, S. Differentiation of movement behaviour in an adaptively diverging salamander population. *Mol. Ecol.* **26**, 6400-6413 (2017).

Steinfartz, S., Weitere, M. & Tautz, D. Tracing the first step to speciation: Ecological and genetic differentiation of a salamander population in a small forest. *Mol. Ecol*. **16**, 4550–4561 (2007).

Caspers, B. A., et al. The more the better – polyandry and genetic similarity are positively linked to reproductive success in a natural population of terrestrial salamanders (*Salamandra salamandra*). *Mol. Ecol*. **23**, 239–250 (2014).

Peakall, R. & Smouse, P. E. GenAIEx 6.5: genetic analysis in Excel. Population genetic software for teaching and research- an update. *Bioinformatics* **28,** 2537–2539 (2012).

Excoffier, L. & Lischer, H. E. L. Arlequin suite ver 3.5: a new series of programs to perform population genetics analyses under Linux and Windows. *Mol. Ecol. Resour.* **10,** 564–567 (2010).

Guo, S. W. & Thompson, E. A. Performing the exact test of Hardy-Weinberg proportion for multiple alleles. *Biometrics* **48,** 361–372 (1992).

Helfer, V., Broquet, T. & Fumagalli, L. Sex-specific estimates of dispersal show female philopatry and male dispersal in a promiscuous amphibian, the alpine salamander (*Salamandra atra*). *Mol. Ecol*. **21**, 4706–4720 (2012).

Valbuena-Ureña, E., Soler-Membrives, A., Steinfartz, S., Orozco-terWengel, P. & Carranza, S. No signs of inbreeding despite long-term isolation and habitat fragmentation in the critically endangered Montseny brook newt (*Calotriton arnoldi*). *Heredity* **118**, 424–435 (2017).

Lourenço, A., Álvarez, D., Wang, I. J. & Velo-Antón, G. Trapped within the city: integrating demography, time since isolation and population-specific traits to assess the genetic effects of urbanization. *Mol. Ecol*. **26**, 1498–1514 (2017).

Kéry, M. & Schaub M. Bayesian population analysis using WinBUGS: a hierarchical perspective. Oxford, UK (Academic Press, 2011).
